# COVID-19 Trend Estimation in the Elderly Italian Region of Sardinia

**DOI:** 10.3389/fpubh.2020.00153

**Published:** 2020-04-24

**Authors:** Mariangela Valentina Puci, Federica Loi, Ottavia Eleonora Ferraro, Stefano Cappai, Sandro Rolesu, Cristina Montomoli

**Affiliations:** ^1^Unit of Biostatistics and Clinical Epidemiology, Department of Public Health, Experimental and Forensic Medicine, University of Pavia, Pavia, Italy; ^2^Osservatorio Epidemiologico Veterinario Regionale Della Sardegna, Istituto Zooprofilattico Sperimentale della Sardegna G. Pegreffi, Cagliari, Italy

**Keywords:** COVID-19, coronavirus, public health emergency, pandemic, SIRD-model

## Abstract

December 2019 saw a novel coronavirus (COVID-19) from China quickly spread globally. Currently, COVID-19, defined as the new pandemic by the World Health Organization (WHO), has reached over 750,000 confirmed cases worldwide. The virus began to spread in Italy from the 22nd February, and the number of related cases is still increasing. Furthermore, given that a relevant proportion of infected people need hospitalization in Intensive Care Units, this may be a crucial issue for National Healthcare System's capacity. WHO underlines the importance of specific disease regional estimates. Because of this, Italy aimed to put in place proportioned and controlled measures, and to guarantee adequate funding to both increase the number of ICU beds and increase production of personal protective equipment. Our aim is to investigate the current COVID-19 epidemiological context in Sardinia region (Italy) and to estimate the transmission parameters using a stochastic model to establish the number of infected, recovered, and deceased people expected. Based on available data from official Italian and regional sources, we describe the distribution of infected cases during the period between 2nd and 15th March 2020. To better reflect the actual spread of COVID-19 in Sardinia based on data from 15th March (first Sardinian declared outbreak), two Susceptible-Infectious-Recovered-Dead (SIRD) models have been developed, describing the best and worst scenarios. We believe that our findings represent a valid contribution to better understand the epidemiological context of COVID-19 in Sardinia. Our analysis can help health authorities and policymakers to address the right interventions to deal with the rapidly expanding health emergency.

## Introduction

The severe acute respiratory coronavirus disease 2019 (COVID-19) disease has been declared as the second pandemic disease of the twenty-century, after the A/H1N1 pandemic in 2009 (1). COVID-19 is a complex human viral infectious disease caused by an RNA virus (genus *Betacoronavirus*), named SARS-CoV-2 due to its 82% similarity with the SARS coronavirus (SARS-CoV) (2, 3). This severe acute respiratory syndrome's common symptoms include: fever, dyspnoea, fatigue, dry cough, and acute respiratory distress syndrome (4). The virus spreads mainly through person-to-person contact via respiratory droplets generated by coughing and sneezing, or even through contaminated surfaces (4, 5). Recent studies based on China showed an increased chance of in-hospital death associated with old age, male sex, and contemporary comorbidities (i.e., hypertension, diabetes, and coronary heart disease) (6–8). Currently there is no available vaccine or a specific antiviral treatment recommended for COVID-19, so the early diagnosis is fundamental to promptly treat the symptoms (9). Furthermore, since infected patients with respiratory complications related to COVID-19 required hospitalizations in Intensive Care Units (ICU), this may be a crucial issue for the National Healthcare System's capacity (10). The negative impact of this disease on the worldwide economy has been discussed and enormous socio-economic losses have been hypothesized, even more than the SARS epidemic (11). Nevertheless, specific economic impact estimation is highly uncertain and seems to be full of gaps, and an accurate estimation could be impossible. All these factors make it difficult for policymakers to formulate an appropriate macroeconomic policy response (12, 13). A global macroeconomic analysis demonstrates that even a contained outbreak could significantly impact the economy and greater investment in public health systems will be the only way to avoid economic disasters (14). The dramatic evolution of COVID-19 resulted in a severe health scenario in China for the first 3 months of 2020 (15). From 20th January the disease spread outside China with two cases in Thailand, one in Japan, and one in the Republic of Korea (16). Five days later the first case was reported in French Republic. The first full-blown outbreak in Europe occurred on 22nd February in Italy (17, 18) and subsequently the disease spread all over the continent and worldwide with about one million cases as of 8th April, 2020 (19). The total number of cases in Italy continues to increase, exceeding 139,000 people, and the number of people killed by COVID-19 (17,669) outweighs the deaths recorded in China (3,259). Using the same data, in Italy ~5,200 beds in ICU (10) are available and 3,693 patients are already admitted in ICU (18). The most affected Italian Region was Lombardy with 53,414 confirmed cases, 9,727 deaths, and 1,257 admitted in ICU (18). Recent studies tried to provide the number of COVID-19 case estimations in different world areas (20). The first official predictive model for the Italian disease spread was published a few days after the first disease occurrence (10). This study assimilates the number of Italian cases increment to an exponential trend and correctly predicted more than 30,000 infectious and 4,000 hospital beds needed during the first 2 weeks of March 2020. The Italian Government put in place extraordinary measures, principally based on limiting contact to contain the virus spread, even before the WHO official declaration of pandemic status on 11th March 2020 (1). The national Decree-Law declared a “lockdown” of all commercial and retail activities, with an exception for basic necessities stores, and movement was limited to only for work activities, health reasons, and urgent needs (i.e., to buy groceries, care for the elderly, or reach one's own house) (21–25). Air and sea transport were subject to the same rule with specific involvement of Italian Islands Regions (Sardinia and Sicily). From 17th March, in order to strengthen the capacity of the Healthcare System to face the emergency, the Government identified funding to increase ICU beds and for the production of personal protective equipment. In addition, the Italian Ministry of Defense has activated an extraordinary procedure for the recruitment of biologists, physicists, chemists, military doctors, physicians, and nurses (26). Following the National regulations, considering the particular condition of Sardinian island and the several problems related to potential patients' transport toward other regions, from 14th March 2020 Sardinian Region ordered the closure of air and sea passenger transport (27). Transport routes will be available only for freight. Given the insulating conditions, the health check of passengers arriving by sea assumes strategic importance. Specific protocols have been put in place in the main Sardinian naval ports, aimed to control the COVID-19 spread (28). After the first SARS-CoV-2 case reported on the 2nd March in the metropolitan area of Cagliari, few cases have been reported all over the island. The first declared outbreak in Sardinia dates back to 15th March in the north of the island (Sassari province) with 35 cases officially reported, when for the first time the intra-hospital contagion involved not only healthcare staff but also patients (28). From that time, the disease spread all over the region (29, 30). This study aimed to investigate the current COVID-19 epidemiological situation in Sardinia based on official reported data. Furthermore, a stochastic model has been applied to estimate the transmission parameters and establish the number of SARS-CoV-2 positive, recovered, and deceased people expected. The present analysis aims to be a valid instrument to help political leaders and health authorities to manage the disease in an island where, if the most appropriate health measures are taken, the epidemic could be more rapidly controlled.

## Materials and Methods

### Study Area

Sardinia is an Italian island located in the middle of the Mediterranean Sea, with a total area of 24,100 km^2^, divided into four provinces [Cagliari (Metropolitan city and Sud Sardinia), Oristano, Sassari, and Nuoro], including 377 municipalities (31). Despite being defined as the oldest civilization in Italy, in most of the Sardinian territory a modern and diversified economy coexists with a still intact natural ecosystem. Sardinia is one of the least populated regions of Italy (32) (69 inh./km^2^) and holds the record for the oldest population in Italy and one of the oldest all over the world (33). The last population census performed by Statistic National Italian Institute (ISTAT) showed that Sardinian people have an average age (46.3 years) older than Italy's national level (44.9 years), and an elderly rate of 212% with respect to the Italian rate of 173% (34). During the last year, a total of 389,614 people aged ≥65 years were living in Sardinia, over a total population of 1,639,591 inhabitants. Most of the elderly population lives in Oristano province (elderly rate = 262%), while Sassari is the province with the lowest elderly rate in Sardinia (194.3%), however still higher than the national average (31). Cagliari is the most populated province with about 780,000 people (including Sud Sardinia), followed by Sassari (500,000), Nuoro (208,000), and Oristano (197,000). Over the island, a total of 32 hospitals are present with a total of 486 ICU available for COVID-19 emergency (172 in the north and 314 in the south), as reported in the official Sardinian Region strategic plans against COVID-19 (35).

### Data Collection

For the purpose of this work, an *ad hoc* case report based on Sardinian COVID-19 cases has been set up. Considering a study period between 2nd and 15th March 2020 (1st period), data about province, city, date of reported infection (dd/mm/yyyy), sex (where available), hospitalization (yes/not), exposition, and contagion type (intra-hospital: yes/not), were collected from official sources (29, 30). Patients reported as SARS-CoV-2 infected have been classified by way of exposure: “From Italian Red Zone” included subjects who arrived in Sardinia from high risk areas (North Italy); “2nd contagious—Red Zone” included subjects living in Sardinia who developed COVID-19 after contact with subjects who arrived from the Italian Red Zone; “2nd contagious” included subjects infected not directly by the Red Zone. Data related to 16th March-−8th April (2nd period) were collected by official sources and used to evaluate the current situation in Sardinia. All Sardinian SARS-CoV-2 positives were laboratory-confirmed by regional accredited laboratories and Istituto Superiore di Sanità (ISS).

### Seasonal SIRD Model Formulation

**Figure 1:**
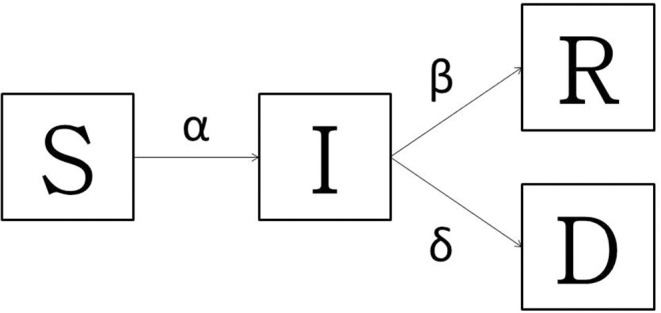
The transmission diagram for Susceptible-Infected-Recovered -Dead model: basic model with death and the recovered. The bold arrows indicate the flow between compartments.

In order to pursue the main goal of this work, the baseline model used was a typical Susceptible-Infectious-Recovered-Dead (SIRD) model, largely used for the so-called “immunizing infections” whose properties are well-understood as fitting well to Italian COVID-19 spread (36, 37). Since no vaccine or population immunity is available, the model accounts for only two outcomes: death or recovery. The all Sardinian population is assumed to be randomly distributed and closer; no births or unrelated deaths are considered. Applying the SIRD model (Figure 1), at any time *t* ≥ 0, the susceptible people *S*(*t*) moved to the infectious *I*(*t*) compartment when they become infected. After an infectious period, the subjects entered in status of recovered *R*(*t*) (successfully immunized) or *D*(*t*) disease-induced death. At any time, the overall population (*N*) is described as *S*(*t*) + *I*(*t*) + *R*(*t*) + *D*(*t*) = *N*(*t*). The model simply keeps track of the number of individuals in each class and those who move from one class to another. The state variables change according to a system of differential equations:

$$\begin{array}{r} S\left(t\right)=\;\frac{dS\left(t\right)}{dt}=\;S_{t-1}-\frac{\alpha }{N}S_{t-1}I_{t-1}\\ I\left(t\right)=\;\frac{dI\left(t\right)}{dt}=I_{t-1}+\frac{\alpha }{N}S_{t-1}I_{t-1}-\;\beta I_{t-1}-\delta I_{t-1}\\ R\left(t\right)=\frac{dR\left(t\right)}{dt}=R_{t-1}+\beta I_{t-1}\\ D\left(t\right)=\frac{dD\left(t\right)}{dt}=D_{t-1}+\delta I_{t-1} \end{array}$$

The infection rate α describes the translation from S to I, so the change in population is equal to -αSI and the transmission process for the non-linear term αSI is describes as:

$$\begin{array}{l} \alpha SI\left(t\right)\;=\;I\left(t\right)*\frac{S\left(t\right)}{N}*\alpha N \end{array}$$

*N* is the population size, α*N* is the number of contacts by the infected per unit time, and the ration $\frac{S}{N}$ is the fraction of these contacts. The infected people could die at rate δ, or recover at rate β. In order to provide a useful instrument to stakeholders, given that the disease is particularly aggressive in elderly patients (38), the amount of the Sardinian population over 60 years who became infected with and died of COVID-19 has been estimated based on Sardinian SIRD models results and tajes into account the infectious rate and lethality by age-classes rate proposed by Istituto Superiore di Sanità (39). The models were stochastically implemented in R-software (Version 3.6, R-Foundation for Statistical Computing, Vienna, Austria); “deSolve” R package was used for implementation and solution of differential equations (40).

### Model Parameterization and Simulation

As underlined by several studies, the main problem of these models is the approximation of the epidemiological parameters (i.e., α, β, δ, and R0), since the actual number of infected *I*_(t)_ people is underestimated or even unknown (20). Furthermore, it is essential to consider the possible confounding role of different disease control strategies by country, which can make a substantial difference in identifying the real number of infected patients (41–44). In a specific population where all individuals are susceptible to infection, R0 represents the average number of secondary cases generated from the introduction of a single infectious case during the infectiousness period. In order to avoid the problem related to R0 classical definition, which strictly depends on α, β, δ rates, the calculation proposed by Anastassopoulou et al. (20), has been applied, considering Sardinian reported cases from 22nd to 29th March 2020. Given the limited data on COVID-19 transmission parameters, and considering the recent incursion of the disease in Sardinia which does not allow for an accurate estimation of the recovered subjects (as the diagnostic assessment of the recovered people takes longer than the diagnostic assessment of infection), three methods have been compared to assess β and δ. First, bibliographic research using PubMed database has been carried out. The key words used alone or in combination were “COVID-19,” “SARS-CoV-2,” “transmission rate,” “mortality rate,” and “SIR model.” The research made available a range of data for each estimate (minimum, maximum, and “most likely” value), useful to perform the probability model (4–10, 15, 20, 41–44). The beta-PERT distribution (45) has been used to generate the distribution that more closely resembles realistic probability distribution of β and δ. Monte-Carlo simulation has been applied based beta-PERT parameter estimation, with 50,000 iterations after a burn-in for convergence of 10,000 iterations. Estimations proposed by this method were equal to 0.116 (min–max = 0.089–0.230) and 0.0012 (min–max = 0.0007–0.0015) for recovery and death rate, respectively. Furthermore, the computation based on corresponding cumulative functions (20) have been applied for both parameters (β = 0.164, 95% CI 0.041–0.187) and δ as 0.059 (95% CI 0.033–0.119). Finally, the rate re-calculation proposed by Baud et al. (44) has been applied on date from the 15th March in Sardinia, showing a recovery rate of 0.154 (95% CI 0.117–0.191) and a death rate of 0.001 (95% CI 0.0008–0.0012). Two scenarios have been simulated: beta-PERT estimation has been applied for the worst scenario while the re-calculated rates have been used to simulate the best scenario for COVID-19 in Sardinia, considering the Italian Government measures and assuming α infection rate halved. For each scenario, the R0 has been estimated based on recovery rate (β) and fatality rate (δ), as usual for SIRD models (36). Giving β and δ, an infected rate (α) approximation can be described by *g* = α*S* − γ, where γ is the inverse of the mean recovery time in days [i.e., average time considered for infection resolution 14 days, γ = 1/14 (4)], thus α becomes a function of the initial susceptible population. The *S*_(t0)_ population has been set equal to = 1,639,591 [Sardinian inhabitants (34)]. By 15th March in Sardinia a total of 77 infectious people [*I*_(t0)_ = 77] were reported, two patients had died [*D*_(t0)_ = 2], and no patients were recovered. The model has been run for 170 days at 1-day interval. The algorithm is run for 100,000 iterations with a burn-in of the first 70,000 iterations. In order to highlight the number of elderly that could be involved in COVID-19 spread in Sardinia, based on both scenarios estimated and region specific disease infectious rate (by age classes) calculated by Istituto Superiore di Sanità (39), different scenarios have been simulated. Considering the lack of data and the absence of specific parameter estimations among elders, the same parameter values of general SIRD models have been used, projecting the specific rates on the estimated infected overall population. Supposing a number of infected subjects, the proportion of infected by age class has been estimated.

## Results

**Figure 2:**
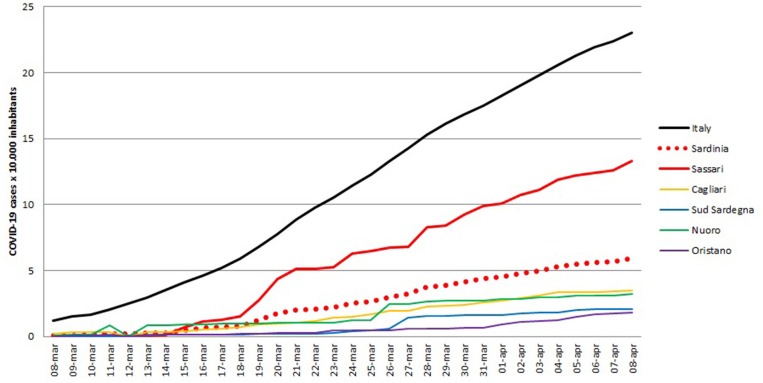
Number of SARS-CoV-2 positive cases for Italy, Sardinia region overall and Sassari province, expressed as number of positive cases × 10.000 inhabitants.

**Figure 3:**
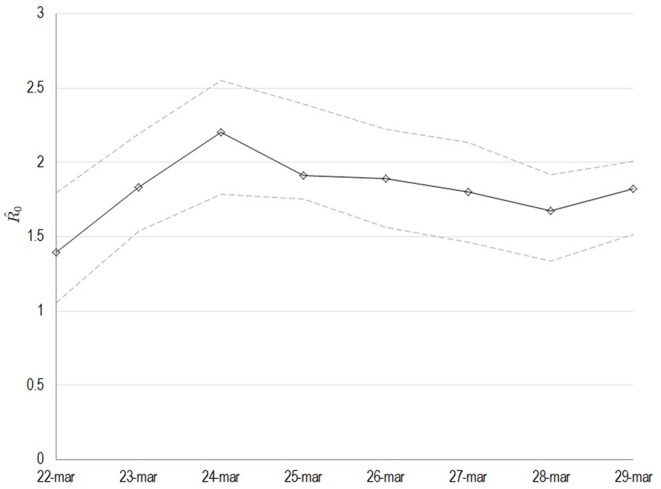
Estimated values of the basic reproduction number (R0) as computed by least squares using a window with initial date the 22nd of March. The solid line corresponds to the mean value and dashed lines to lower and upper 95% confidence intervals.

**Figure 4:**
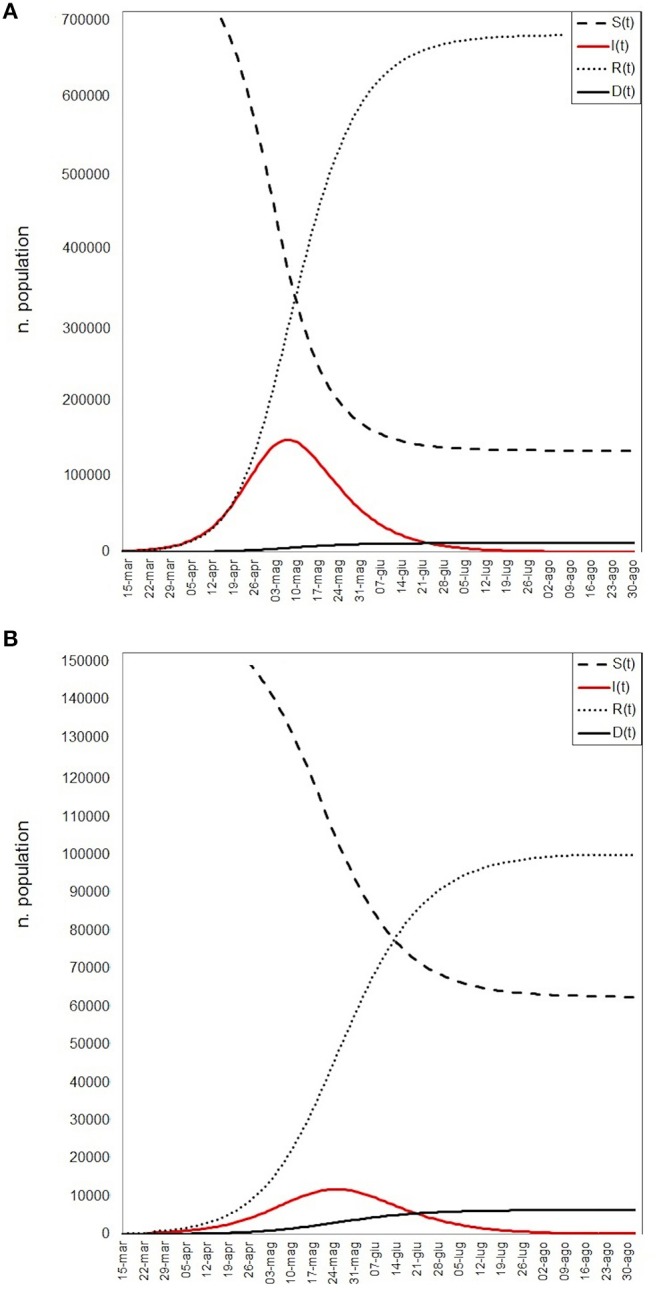
SIRD models simulation: red line represents the infectious (I), continuous black line the deaths (D), little dashed line the recovered (R), large dashed line the susceptible (S) population. **(A)** The worst scenario results, **(B)** the best scenario results.

**Figure 5:**
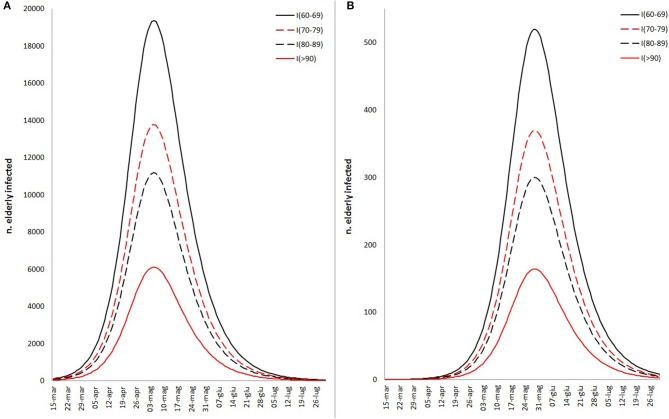
Different fitted scenario estimation about the proportion of elderly infected people by COVID-19, based on worst **(A)** and best **(B)** scenarios fitted by SIRD model and the proportion of Sardinian infectious rate by age classes (60–69, 70–79, 80–89, >90).

All variables used for the main goal of this work are reported in Table 1, by 1st period, 2nd period, and overall. During the time between the first SARS-CoV-2 infection in Sardinia (2nd March) and the outbreak in Sassari (15th March), a total of 77 cases have been officially declared by Sardinian Region. For 68 subjects, data about exposure has been collected: 18 have become infected in the North of Italy (coming back from “Red Zone”) or after direct contact with COVID-19 positive people from “Red Zone,” by out-hospital transmission. A total of 50 subjects contracted the disease by 2nd contagious exposure, not through direct contact with people from “Red Zone,” and by intra-hospital transmission (65% of the overall contagious). The intra-hospital contagious involved only medical staff until the 15th March when, for the first time, intra-hospital infection involved patients. From that time, the number of cases in Sassari province increased dramatically. On 8th April, data expressed as number of cases × 10.000 inhabitants, showed a rate of about 23 in Italy, 5.9 in Sardinia, 13.3 in Sassari, with lower rates having been recorded in the other provinces (Figure 2). In Sardinia 975 subjects have been infected by SARS-CoV-2, of these 112 are recovered with symptoms, 31 are in ICU, and 59 have resulted in death. In Sassari the amount of cases increased 18 times from the first official outbreak to 8th April. As reported in Table 1 and considering the overall data, the number of asymptomatic patients is around 30% and the hospitals (or nursing homes) confirm their critical role as way of spreading contagions between patients (23% of the total). The most affected age classes are 40–49 (20.4%) and 50–59 (21.8%). The mean reproductive number R0 calculated using the data until 22nd March results in 1.39 (95% CI 1.05–1.79) while until 29th March is 1.82 (95% CI 1.51–2.01) (Figure 3). Fitting the worst scenario SIRD model, the R0 has been estimated as 2.23 (95% CI 1.84–2.56). Using the estimated parameter, the trend of Sardinian COVID-19 infection showed an expected peak around the 3rd May 2020 and the number of infected individuals [*I*_(t)_] is about 130,000. At the same date, the estimated number of recovered and thus immunized people is 325,000, while for 8,300 inhabitants the disease would be fatal (Figure 4A). The best scenario fitted using the re-calculated rates showed a lower average of R0 equal to 1.54 (95% CI 1.18–1.97). Under the Italian Government quarantine measures, the expected time for the peak should be later (21st May 2020), with a total number of infected people around 11,500, about 50,000 recovered and a reduced number of deaths (1,800) (Figure 4B). Figure 5 shows the proportion of elderly people (by four different age classes) that could be involved in COVID-19 infection, based on the two different worst and best scenarios. During the first peak time (3rd May 2020) a total of 19,000 people 60–69 years old, 13,600 people 70–79 years old, 11,000 subjects 80–89 years old, and about 6,000 people older than 90 years could be infected by COVID-19 (Figure 5A). At the peak time estimated by the best scenario (21st May 2020), respectively, about 520, 370, 300, and 165 subject by the same age classes has been estimated to be infected (Figure 5B).

**Table 1:** Data related to subject involved in COVID-19 epidemic in Sardinia, by 1st and 2nd period and overall, collected from various official sources, and presented as number (*n*) and percentage (%).

**Variables**	**2nd−15th March [*n* (%)]**	**16th March−8st April [*n* (%)]**	**Overall [*n* (%)]**	**Source**
N. of infectious	77 (7.9)	898 (92.1)	975	(18, 29, 30)
N. of deaths	2 (3.4)	57 (96.6)	59	(18, 29, 30)
N. of recoveries	0 (0)	76 (100)	76	(18, 29, 30)
N. of at home isolation	59 (8.5)	638 (91.5)	697	(18, 29, 30)
N. of hospitalized	16 (14.3)	96 (85.7)	112	(18, 29, 30)
N. of hospitalized in ICU	0 (0)	31 (100)	31	(18, 29, 30)
N. of laboratory tests executed	613 (7.3)	7,880 (92.7)	8,493	(18, 29, 30)
Asymptomatic patients Symptomatic patients	Not available	Not available	295 (30.2) 680 (69.8)	(46)
Exposure				(29, 30, 39)
Intra-hospital contagious	50 (65)	Not available	224 (23)	
Out-hospital contagious	18 (23)		751 (77)	
Unknown	9 (12)		–	
N. of infectious by province				(18, 29, 30, 46)
Cagliari	18 (12.0)	133 (88.0)	151	
Sassari	35 (5.4)	619 (94.6)	654	
Oristano	2 (6.9)	27 (93.1)	29	
Nuoro	19 (28.4)	48 (71.6)	67	
Sud Sardinia	3 (4.1)	71 (95.9)	74	
Lethality rate, by sex				(46)
Male	Not available	Not available	37 (62.7)	
Female			22 (37.3)	
Age of infectious^*^				(39)
0–9			4 (0.8)	
10–19			5 (1)	
20–29			19 (3.9)	
30–39	Not available	Not available	60 (12.2)	
40–49			100 (20.4)	
50–59			107 (21.8)	
60–69			73 (14.9)	
70–79			52 (10.6)	
80–89			42 (8.6)	
≥90			23 (4.7)	
Unknown			5 (1)	

* *Last update 30th March by Istituto Superiore di Sanità. To date, no data by age classes is available from Sardinian Region*.

## Discussion

Considering the WHO Strategic and Technical Advisory Group for Infectious Hazards recommendations about COVID-19, several countries based their disease control strategies on a combination of containment and mitigation activities, to delay major surges of patients and reduce the demand for hospital beds, thereby protecting the most at risk categories (i.e., elderly people and those with comorbidities) (47). Instruments on national risk assessment (i.e., definition of infection period, estimated numbers of infected patients, and hospitalization needed) play a fundamental role in designing a correct health program (41). To date no COVID-19 risk assessment is available for Sardinia region, where the disease control could take advantage as it is an island. As underlined by several previous studies, the most difficult prediction to make is the number of infected patients at the time of the disease peak (10, 20, 48). However, this prediction is of crucial importance to plan appropriate COVID-19 management programs and to calculate the time period at which additional health resources are needed. The present study partially contributes to fill the gaps identified by Bedford et al., based on the WHO national risk assessment about the need to better define the period of infectiousness and transmissibility and to estimate the reproductive number in different regions and countries (41). Based on the COVID-19 Sardinian cases up to 15th March 2020 we estimated the worst and the best scenarios. As expected, changing the parameter estimation (i.e., mortality rate, death rate, and number of contact), the number of expected infected patients, recovered patients, and deceased subjects change drastically, as well as the peak date (20, 36). Based on the parameters resulting from available bibliography, the peak of the disease seems to be early (3rd May 2020) compared to the peak assessed based on the Sardinian parameters re-calculated (21st May). At the same time, the number of infected patients changed from 130,000 to 11,500. It must be considered that it is not obvious, and it will be very unlikely to observe these estimated cases as the number of infected patients that will be officially reported. In fact, the asymptomatic subjects play a key role in disease transmission. As observed by Li et al. (46), 86% of the total COVID-19 infections within China were unreported (95% CI 82–90). If this assessment were valid in our context, a total of about 18,200 (95% CI 13,000–23,400) and 1,610 (95% CI 1,150–2,070) cases should be observed, based on the worst and best scenario, at the peak time, respectively. Considering the last estimation of the Imperial College COVID-19 Response Team, the estimation of infected patients in Italy should be about 9% (95% CI 3.2–26) of the total population (42). If this assessment is applied on the Sardinian population, a total of 140,000 should be or could become infected. Two different methods to calculate/estimate the R0 have been applied. Considering both scenarios, the average R0 calculated seems to be more similar to the best scenario estimation, even if some days reach the worst scenario R0 values. Furthermore, the value of R0 calculated in this study is in line with the findings in the last COVID-19 R0 revision performed by Liu et al. (48). Currently, the most common COVID-19 lethality rate assessment is based on the ratio between deaths and infected people. According to Ghani et al., this method is accurate only at the end of an epidemic, while it can be misleading if, at the time of the analysis, the result is unknown for a not negligible percentage of patients (49). Based on an alternative calculation method, Baud et al. (44) published a re-estimation of the COVID-19 mortality rate as the number of deaths on a given day over the number of COVID-19 infected 14 days before. The study underlines a mortality rate underestimated by 1.5% (95% CI 1.2–1.7) at 1st March 2020, compared to recalculated mortality rate of 15.2% (95% CI 12.5–17.9) outside China. Applying this re-calculation on Italian data from 8th April, the observed lethality rate changes from 12.7% (95% CI 12.5–12.8) to 23.7% (95% CI 23.4–24.1). Essentially, the authors support the measures applied in Italy and Sardinia to control the SARS-CoV-2 spread, which will certainly soon yield encouraging results. From early March, the Italian and Sardinian Governments made a great effort to limit as much as possible the amount of contact between people, therefore limiting the contagion. Several Chinese studies showed that the peak value persistently decreases by reducing contact rate, but may either delay or bring forward the peak by 6.5–9 days (min–max = 5–9). Since the isolation of people can significantly lower the peak and reduce the cumulative number of predicted reported cases, even in an elderly population (38), the results of the present study can suggest that enforcing the restrictive measures can rapidly improve the situation. When the disease arrived in Sardinia, the restrictive measures were already in place, thus the peak of the disease could occur later than in the rest of Italy. Furthermore, it is necessary to consider the particularly low population density of Sardinia (69 inh./km^2^) and that the 27% of elderly people (≥65 years) live alone and isolated (34). Thus, the worst scenario could overestimate the number of cases, since the α parameter is based on the average worldwide amount of contact. While in early stages of the epidemic most of the contagions were intra-hospital, to date the spread of the disease in Sardinia seems to be occurring mostly in hospital and nursing homes (23–41% of total cases, depending on province) (39, 50). Even if this is dramatic for the at risk categories, an appropriate isolation of these cases could drastically suppress the disease spread. This generates a borderline difference between Italy and Sardinia in the average age of the infected/deceased subjects, which hampered the use of national lethality rates in estimating regional lethality. In Sardinia the median age is 83 years old, while in Italy most of the people who died from Covid-19 were 80 years old (50, 51). Although the results partly suggest that the current Sardinian situation is more similar to the best scenario than the worst one, it should be kept in mind that only if a policy of highly restrictive measures is maintained, a further reduction of R0 is achieved. However, several authors consider a long-term disease spread, that may last up to the next 18 months. Given the worldwide economic and social difficulties in maintaining such a high level of restrictions, an adaptive policy needs to be considered: social distancing would only be applied after that confirmed cases admitted in ICU exceeds specific threshold, while this policy will be relaxed when ICU case incidence falls below the threshold. On the contrary, case-based policies of home isolation of symptomatic cases must persist (43). Finally, different studies underline that healthcare staff in work environments with a high risk of infection experienced feelings of anxiety and extreme fatigue and that subjects in quarantine felt negative feelings such as anger and stress. For this reason, although the treatment of the critical infection consequences and the COVID-19 spread containment is the priority, the psychological impact cannot be underestimated and the possibility of offering psychological online support should be considered (52, 53).

## Conclusion

In our opinion, in order to effectively manage the pandemic it is essential to promptly implement extraordinary and combined measures. In this respect, health policy strategies should primarily aim at supporting the healthcare system through the enhancement of both human and instrumental resources and preventing the spread of the infection. Since data on COVID-19 are collected in real time, day by day, by physicians and health authorities, it is not easy, but it is very important to make use of different tools such as SIRD model or graphics trends, with the identification of possible scenarios, to predict how the disease could evolve. In addition, in order to help healthcare professionals to face the increasing workload and to encourage the population to adhere to extraordinary measures such as quarantine, it is important to consider the psychological impacts and provide the most appropriate information in real time. We hope that our analysis will be a useful tool for Sardinian political and health authorities in organizing the most appropriate intervention to better face the pandemic effectively and efficiently.

## Data Availability Statement

Publicly available datasets were analyzed in this study. This data can be found here: opendatadpc.maps.arcgis.com/apps/opsdashboard/index.html#/b0c68bce2cce478eaac82fe38d4138b1.

## Author Contributions

MP, FL, and OF collected the data, designed the model and the computational framework, analyzed the data, and carried out the implementation. MP, FL, OF, and SC wrote the manuscript with input from all authors. SR and CM supervised all planned work. All authors discussed the results and contributed to the final manuscript.

## Conflict of Interest

The authors declare that the research was conducted in the absence of any commercial or financial relationships that could be construed as a potential conflict of interest.
